# Prediction of Domain Behavior through Dynamic Well-Being Domain Model Analysis

**DOI:** 10.1155/2015/931931

**Published:** 2015-08-17

**Authors:** Steven Bosems, Marten van Sinderen

**Affiliations:** Faculty of EEMCS, University of Twente, P.O. Box 217, 7500 AE Enschede, Netherlands

## Abstract

As the concept of context-awareness is becoming more popular the demand for improved quality of context-aware systems increases too. Due to the inherent challenges posed by context-awareness, it is harder to predict what the behavior of the systems and their context will be once provided to the end-user than is the case for non-context-aware systems. A domain where such upfront knowledge is highly important is that of well-being. In this paper, we introduce a method to model the well-being domain and to predict the effects the system will have on its context when implemented. This analysis can be performed at design time. Using these predictions, the design can be fine-tuned to increase the chance that systems will have the desired effect. The method has been tested using three existing well-being applications. For these applications, domain models were created in the Dynamic Well-being Domain Model language. This language allows for causal reasoning over the application domain. The models created were used to perform the analysis and behavior prediction. The analysis results were compared to existing application end-user evaluation studies. Results showed that our analysis could accurately predict success and possible problems in the focus of the systems, although certain limitation regarding the predictions should be kept into consideration.

## 1. Introduction

In recent years, the number of sensors in the world around us has increased, with smartphones, tablets, and desktop computers increasingly being equipped with devices and software that can obtain information about the world around them. Due to the availability of these sensors, context-aware systems have become increasingly popular with developers and users alike, as this type of system promises to enhance the usefulness for the users depending on the context they are in. With an increase in sensors used to observe different context elements that are to be taken into consideration when making decisions comes an increase in observable context, which makes it harder to predict a context-aware system's run-time behavior at design. Domains for which the correct operation of applications is of the highest importance are those of health and well-being. Well-being is the overall feeling of being healthy and happy; well-being applications are aiming to support a user in doing this. This term is not to be confused with health care, where a health care professional defines a treatment plan together with a patient in order to cure a certain ailment. Well-being applications are to be used by an individual without such professional help.

**Figure 1:**
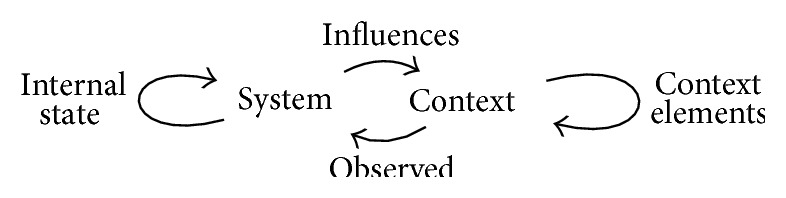
Interaction between system and context.

The type of system regarded in this research is not passive when introduced in the context; rather than only observing context properties, it may also have the ability to influence the context elements, either directly or indirectly. As such, the interaction between the system and the context can be regarded as depicted in Figure 1. The system influences the context, but because of sensor use it is also influenced by this context. Context elements affect each other regardless of system interventions, and the internal system state changes without context changes.

Due to the increase in context-aware systems and the inherent challenges developing them, research in this field is aiming at improving the development process. The four areas of research into the process of creating such systems are requirements engineering [1–4], design [5–9], implementation [10–14], and run-time [15–19]. However, most of the proposed solution directions aim at solving technological problems: how to gather user requirements and translate these into technical solutions, how to deal with pervasiveness, how to manage nearly endless amounts of sensor data, and how to adapt run-time behavior and continuously evaluate whether this behavior is still satisfying the requirements envisioned at design time. Most of these methods, however, fail to take the user's perspective into account, something which is essential for well-being support. Additionally, prediction of the way the system will be behaving at run-time, rather than making run-time predictions of future situations [20] or user needs [21], is challenging, but estimations on the changes in the context once the system is introduced are rarely done. As such, side effects of the system's intervention are not anticipated; rather, they are reacted on after occurrence.

A user-centric, model-driven development process for context-aware well-being systems is discussed by Bosems and van Sinderen [22]. In this previous work, we based application development on context models. In this paper, we will use these context models for context behavior analysis.

Our contributions to the field of context-aware system design are a structured analysis method that can be used to predict system run-time effects on their context at design time. These predictions can be used to improve the system design to prevent undesirable behavior before starting with end-user testing. This analysis method was validated by applying it to three cases.

This work is structured as follows.  Section 2 describes the modeling language used to capture the context of systems, its limitations, and our structured method to perform an analysis on them to predict effects by context-aware systems. In Section 3 we apply this analysis approach to three cases, comparing our analysis results with end-user studies.  Section 4 discusses these results and considers limitations.  Section 5 provides concluding remarks.

## 2. Materials and Methods

This section introduces the Dynamic Well-being Domain Model (DWDM) language [22, 23] and discusses an analysis method to predict the effect of the system on its context.

### 2.1. Dynamic Well-Being Domain Models

**Figure 2:**
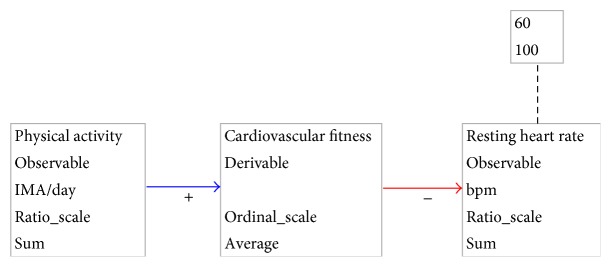
Structure of DWDMs.

When working on the development of software systems, we are concerned with structural aspects, that is, which components can be recognized and how are they related, and behavioral aspects, that is, how will the system react to situations. In the well-being domain, we can recognize similar constructs. To capture both structural and behavioral aspects of the well-being domain, we have created a Domain Specific Language (DSL). Capturing both these context aspects is not possible with current general purpose modeling languages. This DSL was inspired by the Causal Loop Diagram (CLD) notation introduced by Sterman [24]. Where CLDs only provide for notations regarding variables and causal relations between them, we have added properties required to deal with context-awareness and well-being system development.  Figure 2 depicts the structure of DWDMs using a small example. This example illustrates the effects of physical activity on cardiovascular fitness and on the resting heart rate.

*Variables* are elements of the DWDMs that capture the data about the domain. They are represented as rectangles with a maximum of five properties.

*Name*. This is the name of the variable.

*Measurability*. Three types can be found: observable variables, of which the value can be measured directly through sensors currently available, derivable variables, of which the value has to be reasoned about, and controllable variables, which can be directly influenced by the system.

*Unit of Measurement*. Not every variable has a unit of measurement. Most derivable variables will not have a certain unit, whereas all observable variables will.

*Measurement Scale*. The measurement scales defined by Stevens [25] are used here.

*Processing Type*. This property indicates how a variable value should be calculated; a value might stand on its own; the value, when read using a sensor, has a meaning without the need for additional processing. A value may also be dependent on other sensor readings.

A variable may have an associated* norm*, which indicates a range of values which is deemed normal for the general population. Norm values can be obtained from medical literature. In Figure 2, the “resting heart rate” variable has such a norm associated; the normal resting heart rate for the general population is between 60 and 100 beats per minute.

In DWDMs, variables are connected through* causal relations*. Such relations may either be positive or negative. A positive causal relation from *A* to *B* means that an increase in *A*'s value will result in an eventual increase in *B*'s value. No temporal predictions, such as when the change will occur or how long it will take before the change will have reached its full effect, are given; this cannot be predicted reliably for the general population or for an individual user.

**Figure 3:**
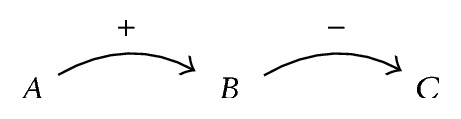
A simple causal path.

When looking at a graph created when connecting variables through causal relations, we can identify two types of constructs. The first is a* path*. A path has a starting and an ending variable and does not contain variable multiple times.  Figure 3 depicts a simple path.

Paths can be used to reason about the way the context captured by the model is affected. If a variable's value changes, all variables down the path are affected too. This is called* forward causal reasoning*. If we are interested in finding out why a variable's value changed, we may perform* reverse causal reasoning*. Here, we follow a path in the opposite direction, from causality target to source. We shall use both methods of reasoning in our analysis method. Looking at Figure 3, an example of forward causal reasoning would be as follows: if *A* increases, then *B* will increase too, which will cause a decrease in *C*. If we were to apply reverse causal reasoning, we would deduce the following: if *C* decreases, this is caused by an increase in *B*, which in turn is caused by *A* increasing. Accurate predictions using causal reasoning become harder when multiple variable changes and causal relations are involved.

**Figure 4:**
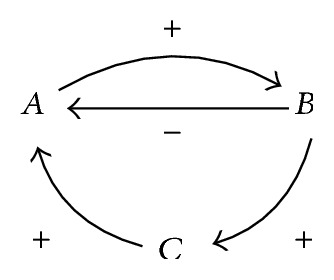
Two causal loops.

The other construct created using causal relations is the* loop*. A loop is a path that starts and ends in the same variable. Loops that have an even (or zero) number of negative causal relations are called* reinforcing*; a loop with an odd number of negative causal relations is called* balancing*. The former type models exponential change; the latter models a stable situation. A reinforcing loop may also be balanced by influences from causal relations from outside the loop itself.  Figure 4 shows an example of a causal graph with two loops. The loop *A*-*B*-*A* is balancing; if *A* increases, this causes an increase in *B*, resulting in a decrease in *A*. On the other hand, the loop *A*-*B*-*C*-*A* is reinforcing; an increase in *A* causes *B* to increase, increasing *C*, which increases *A* again, resulting in additional, continuous increase.

### 2.2. DWDM Limitations

The DWDM language, although deemed suitable for the capturing of the well-being domain, does have several limitations.

Firstly, the modeling language has three types of measurability, whereas adding more could be useful. For example, while some information can only be obtained by directly asking the user, these variables are currently denoted as “observable,” while a term such as “askable” would be more suitable.

Secondly, the modeling language does not allow for the modeling of temporal aspects, which limits the ability to reason about certain timed elements of the domain, such as the time required for effects to be noticeable and how long these effects will remain.

Finally, the DWDM language does not allow for “alternative” constructs; not all relations in the well-being domain are always true; sometimes different relations may take place, depending on domain variables.

### 2.3. Method of Analysis

Our context behavior prediction method is based on a set of questions about an application specific DWDM. Through this structured method, we gain insights into the goals of the application and the way the designers intend to achieve these goals. Knowledge regarding the latter allows us to predict whether the application under design will actually achieve the goals or that problems might arise during execution. If such potential problems have been identified, the system design can be altered to prevent them.

**Figure 5:**
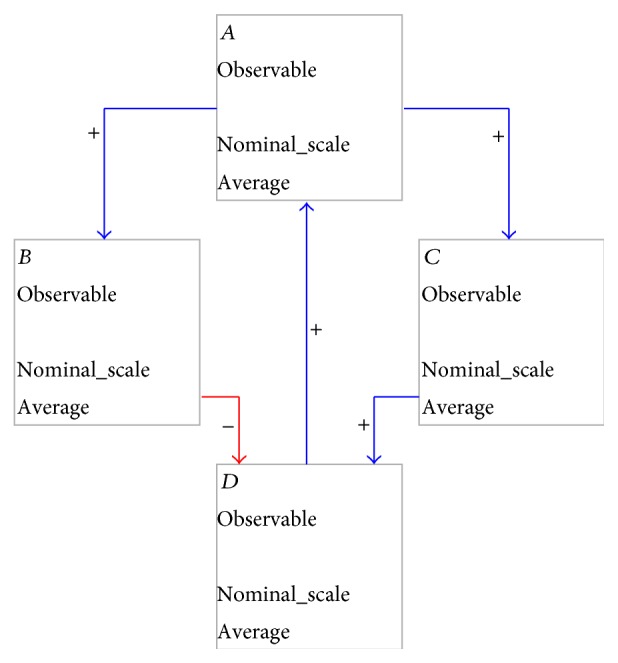
DWDM with four variables and contradicting causal relations.

**Figure 6:**
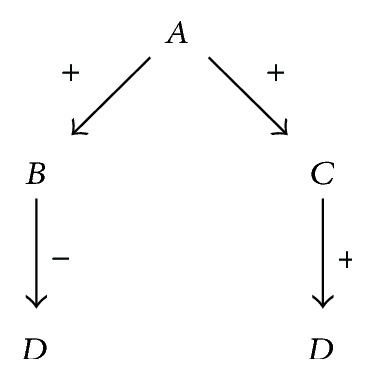


The questions that should be answered for an application specific DWDM are the following:

(1) What is the goal of the application? Which variables are to be increased/decreased? Every well-being application has a* goal*. Such a goal is realized by increasing or decreasing the value of one or more of the domain variables. By identifying this goal, the underlying application logic can better be explained. The goals of the application are represented as goal variables in the domain model.
(2) Which variables are to be affected by the application in order to satisfy the application's goal? At design time, the* means*, that is, the variables that are to be affected in order to attain the application's goals, are decided upon. Means may be either direct (“increase *A* to attain goal *B*”) or indirect (“alter *A*, such that *C* changes, which allows us to attain goal *B*, *C* being the indirect means”). The direct means are those variables which are directly influenced by the system; indirect means are affected by direct or other indirect means, such that the goal variable's value changes. It should be possible to create a path from the means to the goal variable(s). Means for which this is not possible cannot contribute to the application's goal.
(3) Which variables will be measured by the application? In a DWDM, observable variables can be read directly by sensors. We must determine whether all the observable variables in the domain model are indeed monitored or whether they serve another purpose for the application.
(4) Are goals and means variables directly related? If goals and means variables are directly related through a single causal relationship, rather than through indirect relationships, achieving the goal will be easier and potentially undesired side effects can be minimized. The same is true if a goal variable can be directly influenced; that is, the goal and means variable are the same.
(5) Which variables will be directly affected by changes in the means? If the application is introduced in the domain, it will affect context variables. By identifying which variables are directly affected, potential side effects can be predicted.
(6) Which variables are indirectly affected by the changes made? As the domain model may contain more variables than those affected by the system, we want to know which variables are influenced in total. To do so, we draw a tree by exploring the domain model in a depth first search fashion. Loops are not modeled, and if a variable already exists in the tree, it is captured again.
  For example, observe the following DWDM in Figure 5. If *A* were a means for achieving goal *D*, the tree of affected variables would be as in Figure 6.
  As can be seen in Figure 6, the *D* variable appears twice in this tree, and no loops exist. In these trees, we are only interested in the causal relations; the variable properties are not modeled.
(7) Are there variables in the domain model that are not in the tree? Some variables might be part of the domain, influencing the context of the user. However, in some systems, not all of these are monitored or controlled. Not using these variables, however, may have effects on the user's experience of the application.
(8) Are there contradicting effects? Observing the DWDM provided in question (6), we see that an increase in *A* will cause an increase in both *B* and *C*; however, this results in different changes for *D*, depending on whether the causal relation *B*-*D* or *C*-*D* is regarded. Such situations are contradicting. The result of a contradicting situation is unpredictable; as no temporal aspects are modeled, we do not know which change will occur first and which change will occur second or if they occur simultaneously. Depending on the order of effects, one of the causal relations may be prevalent over the other, or effects of either one are negated. In the case of the example mentioned here, the effect of increasing *A* may be either an increase in *D*, a decrease in *D*, or no change at all.
(9) Can contradicting effects be prevented by using other means? If other variables are affected, but the goal still is attained, can conflicts be avoided?
(10) Which loops can be identified? Loops in DWDMs indicate exponential growth or a stable situation, modeled by reinforcing or balancing loops, respectively. Exponential change is rarely desirable.
(11) Are reinforcing loops compensated by balancing loops? As discussed, a reinforcing loop models exponential growth. Situations like this are both highly unlikely to exist and undesirable in the context of well-being systems. As such, attempts should be made to prevent them from occurring. One way of doing so is by having one or more of the reinforcing loop's variables consisting in a balancing loop. This balances the reinforcing loop's effect.
(12) Observations: after following these steps, we formulate an overall observation of the application DWDM, naming observations not captured by the questions.

When all steps have been taken, we can predict certain application behavior and identify potential pitfalls as follows:

1. The application should not have too many goals, as this indicates a lack of application focus.
2. An increased number of means allows the application to achieve its goal through several different ways. This can be regarded as a positive property.
3. As with the increased number of means, an increased number of measured variables indicates the possibility to choose from the data collected. If data collected through one source proves insufficient or unreliable, additional sources can be used.
4. Goals and means that are directly related have a close connection. Increasing distance between means and goals may reduce the strength of causal relations between them.
5. Means that are to affect user behavior through persuasion and feedback are more likely to fail than means that can directly be affected by the system.
6. By creating an overview of all variables affected by or affecting the system, we can identify which variables are* not* affected; the system does not influence them, but they are part of the system's context. Although this might not be a problem, these unaffected variables do affect the domain and are influenced through means not included in or measured by the system; the choice not to affect them might have negative consequences on the effect of the application.
7. Contradicting causal relations may cause unanticipated results due to the temporal effects of causal relations not being documented. Such resulting behavior may include changes caused by one relation immediately being negated by another or being negated after a period of time.

## 3. Application of Analysis Method

Using the analysis method proposed, three applications were analyzed: the TNO/SWELL Fishualization (Activity Board) [26], the Philips/SWELL mBeats application [27], and the Roessingh Research and Development Activity Coach [28]. The causal models for these applications were created through analysis of the application's documentation and interviews with developers or experts. After performing the analysis, the results, that is, the identified success factors and potential pitfalls, were compared with user studies that have already been performed for the applications.

### 3.1. Domain Model Creation

Using the DWDM language, domain models can be constructed that are specific for a single system or application or more general to be reused for multiple development projects. The latter was done by Bosems et al. [29]. This overall well-being model was verified to be correct through questionnaires answered by seven participants and interviews with five experts in both domains. It is possible to derive application specific domain models from this overall domain model.

For the cases discussed in this section, the creation of application specific domain models is done by researching available system documentation. In this documentation, we have looked for the goals the systems aimed to fulfill, how the designers of the systems wanted to achieve this goal, that is, what means were available, and what other domain properties were deemed related. This information was found in unstructured, natural text. Using the overall well-being model cited earlier, the found information was mapped to variables in the model, and paths were created between them. This resulted in connected domain models for all three cases.

### 3.2. TNO/SWELL Fishualization

The TNO/SWELL Fishualization offers a way of providing visual feedback to a group of employees regarding their working habits. The tool consists of a small application that is installed on the user's desktop computer. It tracks keyboard and mouse activity, the applications opened (Microsoft Word, PowerPoint, Outlook, et cetera), and how often switches are made between these applications. The data from all the installed client applications is aggregated and visualized on a monitor at a central location, such as a coffee room. This allows for comparison of working style between colleagues. The idea behind the system is both to improve personal insight in working habits and to initiate conversations among colleagues about experienced work stress.

#### 3.2.1. DWDM Analysis

The Fishualization aims at decreasing the users' experienced stress in order to improve their mental well-being; the goal variable is “mental well-being,” with “feedback” and “department feedback” being means. In addition, the social interaction among coworkers is to be improved, as to encourage conversations about stress and how to prevent it. This is derived from the fact that “department feedback” is to increase, increasing “social interaction,” which should increase “mental well-being.”

The current design of the application provides users with feedback about their work, giving the user an overview regarding his/her computer behavior (“computer activity” is the only measurable variable). Stress related to computer work is recognized; means of measuring this are, among others, keyboard and mouse usage and application switches. The application does not aim to affect all of these stressors, only to provide increased “awareness” and increased “information support”. The application design is based on the assumption that providing feedback regarding the current working conditions is enough to reduce the user's experienced stress and result in conversation among colleagues. No feedback is provided on how to reduce the experienced stressors or how to cope with stress, that is, how to recover, while at work or during free time it only provides the user with a mirror regarding the current situation. Because of this, the application might be interesting to the user at first due to the novelty; however, in the long run it might not be enough to keep the user engaged. Additionally, the application does not value the amount of work/computer input performed. As such, the user is only informed of what the objective values of the perceived stressors are, not whether this is deemed high, low, or average.

**Figure 7:**
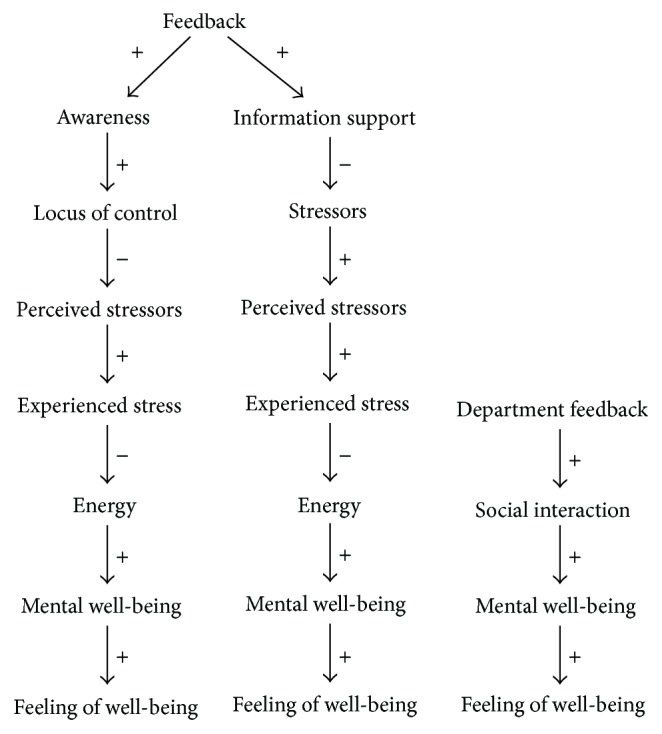
Variables influenced by the TNO/SWELL Fishualization when introduced into the context.

The way context variables are affected by the system can be found in Figure 7. The means of the system are providing feedback and improving department feedback. No variables that are identified in the DWDM are unaffected. No conflicting relations were found.

The application might not succeed because of the following causes: (i) if the provided feedback does not align with the user's expectations, this may cause the provided feedback not increasing the user's locus of control and so will not help to reduce perceived stressors, (ii) if department feedback is not picked up by colleagues, this might not lead to additional social interaction, and (iii) the increase of feedback and awareness could contribute to increasing experienced stress because the user gets confirmation that s/he is indeed busy. However, as the application works through two separate means of user interaction, chances of either one of these succeeding are increased.

#### 3.2.2. User Study

The target of the Fishualization was to increase both the users' and their colleagues' insight in their well-being and levels of working pressure. However, a user study among five participants of a Dutch high-tech company showed that the social interaction that was aimed for was lacking [30]. Users did report an increase in their own insight of their energy levels, but the fish tank did not result in additional conversation and discussion regarding stress and stress prevention among colleagues. Similar results were obtained among 14 employees of an independent Dutch research company. Here, especially insights into personal working patterns and well-being at work improved. Group awareness and social interaction with colleagues, however, remained at the same level.

#### 3.2.3. Comparison

When analyzing the Fishualization, it became apparent that the application influences the user through two distinct means; firstly, the user as an individual is provided with feedback; secondly, the group of users is given overall feedback. The chance for the application to succeed in its goal was estimated to increase because of this, with the remark that department feedback might not lead to an increase in social interaction.

Comparing our predictions to the user experience reports, we find that users indeed did not engage in more talk about their perceived work stress with colleagues. However, due to the duality of feedback, users did receive feedback on their own work, which did result in a feeling of better insight into their energy levels.

### 3.3. Philips/SWELL mBeats

Rather than focusing on the mental well-being of the user, the Philips/SWELL mBeats application wants to improve a user's cardiovascular fitness [27]. The system consists of two parts: a smartphone application and a heart rate sensor that is to be worn on the wrist at all times. The application can display a target heart beat for the user to achieve in order to improve his/her fitness. If the user's heart rate is within this range, s/he is rewarded points (“mBeats”).

#### 3.3.1. DWDM Analysis

The mBeats application aims to increase the user's physical well-being, that is, increase the “physical well-being” variable. It will attempt to provide the user with feedback, that is, increase the “feedback” variable, as to make him/her be active more, increasing their heart rate. “Heart rate” is the only variable that is being measured by the system. Both body composition and cardiovascular fitness are expected to improve, as an increased value for “physical activity” will increase both values for “body composition” and “cardiovascular fitness,” which both have a positive effect on the user's overall feeling of well-being. However, the application is only intended to provide the users feedback about the current and past levels of their heart rate; the application does not influence the user's self-efficacy, so the user will not know whether s/he has performed the activity properly. This which could result in a decline in motivation, which will cause a decline in physical activity as well. Additionally, as the user's activity is not measured (the focus of the application is solely on heart rate), the user might wonder how the cumulative heart rate was calculated, that is, which activities were undertaken to achieve said heart rate.

**Figure 8:**
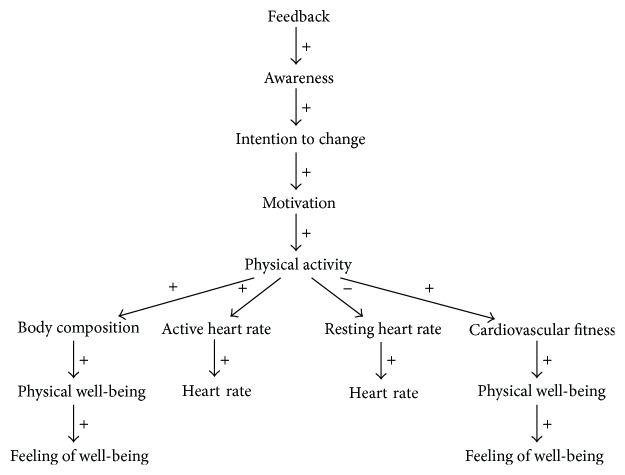
Variables influenced by the Philips/SWELL mBeats application when introduced into the context.

The way the application is designed to influence context variables can be seen in Figure 8.

There are a few possible causes for the application not to succeed: (i) the application relies on the user's self-efficacy, as is the case with most physical well-being support systems, but this variable is not affected by the system, causing the user's motivation to decline, (ii) if the user does not understand the provided feedback, s/he will not engage in activities that increase his/her heart rate, and (iii) if the heart rate measurement is inaccurate, feedback will be inaccurate.

#### 3.3.2. User Study

van Dantzig et al. [27] document the requirements, design process, and user study and evaluation of the Philips/SWELL mBeats application. The experiments performed to evaluate the application primarily aimed to see the effect of providing the users with feedback that included historic data or solely providing them with their current heart rate. We can, however, still draw conclusions about the overall working of the application from the user evaluations. Although participants mentioned that they liked the idea of the application, some remarks were made.

Firstly, the users noted that only providing feedback regarding current heart rate was insufficient to keep them motivated; they would have liked more feedback on how to achieve the heart rate required for cardiovascular fitness improvement.

Secondly, users did not know which activities contributed to getting their heart rate to the right frequency, as the application did not perform activity tracking or recognition. Most users would have liked to be provided with an overall view of the undertaken activity throughout the day.

Thirdly, as the application did not aim at affecting the user's self-efficacy through encouraging feedback, users lost confidence in their ability to meet the applications target throughout the test period. Users mentioned that they would have liked motivational messages from the application.

Fourthly, due to connectivity issues between the measuring device (Mio Alpha heart rate sensor) and the storage and processing device (smartphone), data was frequently lost, causing annoyance with the user.

Finally, half of the participants would have liked more feedback regarding their activity, such as “speed, distance, and calories burned,” suggesting GPS tracking for this purpose.

#### 3.3.3. Comparison

The primary problem found in our analysis was that the mBeats application did not affect the user's self-efficacy. This was reflected by the user evaluation study as being the main reason for people not liking the application; users wanted to be provided with feedback and encouraging messages regarding their activity but did not receive these. Due to the feedback messages being unclear, the self-efficacy of some users even declined.

Another anticipated part of the application design was the lack of activity recognition and tracking. Users indicated that this would be useful as it would provide additional insight into the way the application measured their activity.

Not anticipated by our analysis were the requirement from users regarding certain user interface elements and proper connectivity between sensor and storage device.

### 3.4. RRD Activity Coach

Like the mBeats application, the Activity Coach aims to improve the user's physical well-being. In contrast, the Activity Coach is targeted at patients suffering from Chronic Obstructive Pulmonary Disease (COPD), which eventually causes a decrease in physical fitness due to a decrease in physical activity. The Activity Coach guides its users to spread out physical activity over the course of the day, teaching them not to be too active in the morning, as this would result in a lack of energy in the afternoon, but overall being increasingly active to improve endurance. To measure the user's activity, the Activity Coach uses a triaxial accelerometer, the sensor data of which is used by a smartphone application. Unlike the other two applications, the Activity Coach was designed to be part of a teletreatment intervention, being provided and explained to the user by a health care professional [28].

#### 3.4.1. DWDM Analysis

The goal of the Activity Coach is to increase the user's physical activity, that is, increase the “physical activity” variable's value, in order to improve the user's physical well-being, increasing the value of the “physical well-being” variable. The means through which this is to be achieved is by observing the user's physical activity and by providing feedback regarding historical activity and improving the user's self-efficacy, that is, increasing the “feedback” and “self-efficacy” variables. Due to this duality of feedback, the chance of succeeding in influencing the user is increased. The positive causal relation from “feedback” to “self-efficacy” is specific to this application; the application DWDM adapted to reflect this; the overall well-being DWDM on which this application DWDM is based does not contain this relation. When looking at the variables that should be influenced by the application, we find that there are no variables being unaffected. Chances of application success and long-term adherence are therefore high.

**Figure 9:**
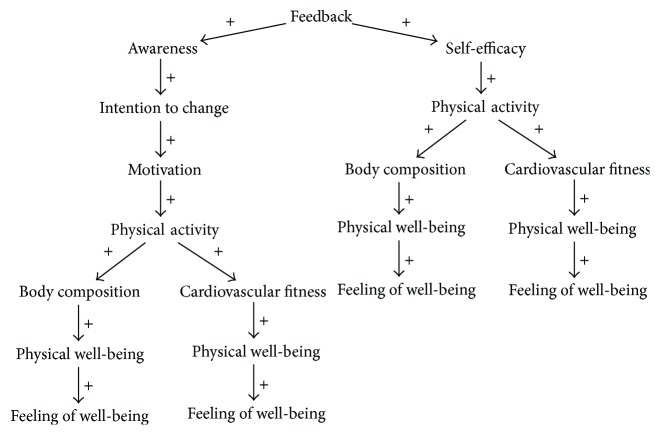
Variables influenced by the RRD Activity Coach when introduced into the context.

The Activity Coach's way of affecting its context is shown in Figure 9.

The Activity Coach might not achieve its target if the following occurred: (i) the user does not correctly interpret the provided feedback, which might not increase the user's levels of activity or his/her self-efficacy, (ii) if the activity is measured inaccurately, caused by incorrect detection of movement by the accelerometer, the provided feedback will be inaccurate too, or (iii) the user cheats the device by putting it down when s/he should not be active or shaking it to simulate activity. This way of cheating the device was not possible in the Fishualization and the mBeats systems, due to the sensor technology used. However, as this application is issued to the user through a health care professional, and it is to be used as part of a larger treatment plan, such scenarios are unlikely.

#### 3.4.2. User Studies

The Activity Coach has been under research for a number of years. One of the first evaluation studies performed was discussed by op den Akker et al. [28]. In this work, the viability of teletreatment for patients suffering from COPD is evaluated. The Activity Coach, as evaluated by us, was part of a larger system that was to support the user and the feedback partially being presented through a web portal and provided by health care professionals. The system overall was accepted by the users; however, no detailed discussion is provided by the authors. The authors do note that effective coaching is highly important to the success of the system.

Tabak et al. [31] apply the Activity Coach, not the entire telemedicine platform, to the case of COPD patient treatment. 21 patients were provided with the application, 7 males and 14 females. Evaluation showed that usability of the application was good to excellent. No evaluation regarding the improvement of physical well-being was performed but was anticipated for future work.

Tabak et al. [32] evaluated the impact of providing the user with real-time motivational cues. Results from 14 patients were evaluated. The users were provided with the motivational cues every two hours; the application tries to motivate people to have the optimal amount of physical activity daily. Over the intervention period, participants showed a significant increase in activity, while especially responding to discouraging feedback cues. The authors note that the period over which the experiment was executed, four weeks, is short for behavioral changes.

#### 3.4.3. Comparison

When analyzing the Activity Coach, we did not anticipate problems with the application, other than potential usability issues. Comparing our analysis with the various user studies performed, this prediction was confirmed. Users rated the application as highly usable; the feedback results in an increase of physical activity over the trial period. It should be noted that the setting in which the Coach was tested (teletreatment of COPD patients) did differ from the user study settings for the other applications.

## 4. Results Discussion and Limitations

This section discusses the results obtained in our experiments, lists lessons learned, and comments on some limitations regarding our method.

### 4.1. Results Discussion

In this work, the DWDMs of three context-aware well-being systems were analyzed according to a 12-step checklist. These DWDMs were constructed using the design documentation that was available. The analysis results were presented in this paper in summarized form.

From the analysis, it became apparent that all three systems provide their users with feedback with the intention to increase awareness regarding the current situation. For the two physical well-being support systems (the Philips/SWELL mBeats and the RRD Activity Coach), such increased awareness is to increase the user's intention to change; for the mental well-being support system (the TNO/SWELL Fishualization), increased awareness was to increase the user's locus of control. The latter system affected two context variables in order to influence the user; the other two systems influenced one variable.

Both the Fishualization and the mBeats application received critical user responses. For the former, this was caused by a lack of response to the centralized display of coworker working habits; the latter did not take self-efficacy into account. The issue with the mBeats application was identified through our DWDM analysis.

Our method consists of twelve questions that are to be used to analyze an application specific DWDM. Although this is a good start, this list of questions may need to be extended in the future to provide a more complete overview of the context behavior. Examples of such extensions could include the effect of the total number of application domain model variables, the result of an increased number of paths in the model, and how the path length from the affected variables to the system goals influences the system's effectiveness. Norms should be researched for such questions.

In all three of our experiments, the DWDM-based analysis closely matched the results obtained from end-user evaluations. In our opinion, the three applications that were analyzed are representative of the current state of the art of context-aware well-being systems. However, more systems should be analyzed to further validate the reliability of our method.

Although our method was designed and validated for the domain of well-being systems, a similar analysis can be performed for any system that is context-aware. The only requirement is that a causal domain model of the context can be created using a language such as DWDM.

### 4.2. Lessons Learned

In the process followed to create the application specific DWDMs, analyzing them and predicting the application's behavior from this analysis, lessons regarding the methods and artifacts were learned.

Firstly, our experience with the creation of the domain models for the three analyzed cases was positive. By using an overall well-being domain model and removing those elements not mentioned in the documentation of the specific cases, application specific DWDMs were easily created. This would have been more difficult if no overall domain model could have been used; as this overall model contains validated relations, the relations in the application domain models can also be assumed to be correct. If these models were created from scratch, they should have been validated by domain experts before they would have been usable for analysis.

Secondly, the analysis process followed to predict application behavior was relatively easy. Answering the twelve questions posed in Section 2.3 was straight forward, given a DWDM of the application. Drawing conclusions from these answers, however, does require in-depth knowledge of the application documentation. For example, only by studying the documentation, it was known how the Philips/SWELL mBeats application was to influence the user, resulting in the conclusion that this way of working was potentially flawed. As such, it can be said that the analysis of the DWDMs and the prediction of behavior cannot be performed fully without the documentation and design rationale of the application. However, without the analysis method proposed in this work, such a prediction would not have been possible; analyzing design documentation in a structured way is highly complicated. The full analysis process including the creation of application specific DWDMs took less than 3 hours per case studied.

Thirdly, in our analysis method, we look at several elements of DWDMs, such as means and goal variables, the presence of loops and the type of loop, the way variables are related, and whether contradicting paths through the DWDM can be found. While performing the analysis and deriving conclusions, we found that most information required to make predictions could be obtained from the trees made by answering question 6 of our method. Although this tree does not contain loops, it is easier to follow and analyze than when looking at the original graph structure of the domain model. Answers regarding the loops were taken into account when formulating the predictions, but following the reasoning process from means to goal variables was easier by observing the trees.

Finally, the results obtained from our analysis method and from the user evaluation studies are highly similar. One might argue that our method is therefor of little added value. However, as it is possible to analyze the DWDM with relative ease, predictions can be made faster than when using traditional evaluation methods, such as end-user studies. With these predictions, the application, while being still under development, can be altered to prevent problems identified by the analysis.

### 4.3. Limitations

Although our method has predicted results of applications correctly, it does have certain limitations. These limitations are primarily caused by the input models used.

Firstly, it is not possible to predict the effect of different user interaction and feedback strategies. It is possible to identify which variables the feedback should concern, but the messages and method are to be defined and evaluated by the application developer.

Secondly, quality properties cannot be captured in DWDMs and as such cannot be reasoned about using our analysis method. Reliability, efficiency, and usability are all highly important to users of well-being applications and services; if these are not satisfied to the levels desired by the user, s/he will stop using the application. We have seen reliability issues occurring in the Philips/SWELL mBeats user evaluation, causing issues in the user experience. Additionally, the user interface provided to the user should align with what the user is expecting of the application, providing the user with the information s/he needs to perform the intervention suggested by the system.

Thirdly, as no temporal information is captured in the DWDMs, no predictions regarding the timing of effects can be made. This would be beneficial when dealing with contradicting causal relations.

Finally, security and privacy aspects of the application under development cannot be represented in DWDMs and so will have to be reasoned about through other methods. As the domain considered consists of highly personal and private data, such requirements are important when aiming for large scale user adoption; users have to trust the application and related services, or they will not use them.

## 5. Conclusions

With an increase in available context information comes an increase in complexity of the design of systems that utilize this information. Predictability of application behavior is important, especially in the well-being domain. Current research primarily focuses on solving technological problems, neglecting the user's perspective.

In this paper, we have introduced a modeling language that can be used to capture the characteristics and structure of the well-being domain. Using models of the well-being domain expressed in this language, we proposed an analysis method that allows us to predict the effect of a context-aware well-being application in its context once it has been introduced in the well-being domain. We analyzed a selection of existing applications using our method and compared the analysis results to the applications' end-user evaluations.

Our contribution to the field of context-aware well-being system design is a method to analyze the domain model of the context on which the application is to act and predict what the effects of the application on this context will be. By comparing this prediction to the intended behavior, possible mistakes can be identified and mitigated at design time, rather than finding them at run-time.

Based on the comparison with the user experience reports, we can conclude that our predictions regarding the context behavior were accurate. We managed to identify potential problems and establish the grounds on which the applications would or would not succeed.

Despite our ability to predict the functional aspects with regard to the context-aware well-being applications, nonfunctional properties could not be anticipated. User interface design, reliability, and security requirements of the applications and context cannot be captured in a DWDM and as such cannot be taken into account in our analysis.

## References

[1] Jorgensen J. B., Bossen C. Requirements engineering for a pervasive health care system.

[2] Salifu M., Yu Y., Nuseibeh B. Specifying monitoring and switching problems in context.

[3] Sawyer P., Bencomo N., Whittle J., Letie E., Finkelstein A. Requirements-aware systems: A research agenda for RE for self-adaptive systems.

[4] Sutcliffe A., Fickas S., Sohlberg M. M. (2006). PC-RE: a method for personal and contextual requirements engineering with some experience. *Requirements Engineering*.

[5] Hendrich N., Bistry H., Adler B., Zhang J. User-driven software design for an elderly care service robot.

[6] Lane N., Mohammod M., Lin M. BeWell: a smartphone application to monitor, model and promote wellbeing.

[7] Mirkovic J., Bryhni H., Ruland C. M. A framework for the development of ubiquitous patient support systems.

[8] Muñoz J., Pelechano V., Fons J. Model driven development of pervasive systems.

[9] van Sinderen M. J., van Halteren A. T., Wegdam M., Meeuwissen H. B., Eertink E. H. (2006). Supporting context-aware mobile applications: an infrastructure approach. *IEEE Communications Magazine*.

[10] Dey A. K., Abowd G. D. The context toolkit: aiding the development of context-aware applications.

[11] Henricksen K., Indulska J. (2006). Developing context-aware pervasive computing applications: models and approach. *Pervasive and Mobile Computing*.

[12] Nuseibeh B. (2001). Weaving together requirements and architectures. *Computer*.

[13] Reichherzer T., Coffey J., Gonen B., Gillett I. Knowledge modeling in the health care domain to support software development & maintenance.

[14] Seyff N., Graf F., Grünbacher P., Maiden N. (2008). Mobile discovery of requirements for context-aware systems. *Requirements Engineering: Foundation for Software Quality: 14th International Working Conference, REFSQ 2008 Montpellier, France, June 16-17, 2008 Proceedings*.

[15] Alférez G. H., Pelechano V. (2012). Dynamic evolution of context-aware systems with models at runtime. *Proceedings of the 15th International Conference on Model Driven Engineering Languages and Systems (MODELS '12)*.

[16] Cheng B. H. C., Sawyer P., Bencomo N., Whittle J., Schürr A., Selic B. (2009). A goal-based modeling approach to develop requirements of an adaptive system with environmental uncertainty. *Model Driven Engineering Languages and Systems: 12th International Conference, MODELS 2009, Denver, CO, USA, October 4–9, 2009. Proceedings*.

[17] Coutaz J., Crowley J. L., Dobson S., Garlan D. (2005). Context is key. *Communications of the ACM—The Disappearing Computer*.

[18] Lee J., Garduño L., Walker E., Burleson W. A tangible programming tool for creation of context-aware applications.

[19] Welsh K., Sawyer P., Bencomo N. Towards requirements aware systems: run-time resolution of design-time assumptions.

[20] Chen G., Chen L. (2015). Augmenting service recommender systems by incorporating contextual opinions from user reviews. *User Modeling and User-Adapted Interaction*.

[21] Khattak A. M., Usman A., Lee S. (2015). Ontology based context fusion for behavior analysis and prediction. *Smart Homes and Health Telematics: 12th International Conference, ICOST 2014, Denver, CO, USA, June 25–27, 2014, Revised Papers*.

[22] Bosems S., van Sinderen M. J. Modeldriven development for user-centric wellbeing support: from dynamic well-being domain models to context-aware applications.

[23] Bosems S., van Sinderen M. J., Meersman R., Panetto H., Mishra A. (2014). Models in the design of context-aware well-being applications. *On the Move to Meaningful Internet Systems: OTM 2014 Workshops*.

[24] Sterman J. D. (2000). *Business Dynamics: Systems Thinking and Modeling for a Complex World*.

[25] Stevens S. S. (1946). On the theory of scales of measurement. *Science*.

[26] Koldijk S., Boertjes E., Verberne S., Wiertz L., Versloot C., Schavemaker J. (2013). COMMIT/SWELL D3.10 SWELL@work applications. http://www.swell-project.net/results/deliverables.

[27] van Dantzig S., Thomassen I., Speelpenning T., Bonomi A. (2013). COMMIT/SWELL D5.4a evaluation of pilot study. http://www.swell-project.net/results/deliverables.

[28] op den Akker H., Tabak M., Marin-Perianu M. (2012). Development and evaluation of a sensor-based system for remote monitoring and treatment of chronic diseases—the continuous care & coaching platform. *Proceedings of the 6th International Symposium on eHealth Services and Technologies, EHST 2012, Geneva, Switzerland*.

[29] Bosems S., Koldijk S., Achterkamp R. Dynamic Domain Model for Well-Being. http://wwwhome.ewi.utwente.nl/~bosemss/2015/caas/wellbeing.png.

[30] Schavemaker J. (2014). COMMIT/SWELL D3v1.7.

[31] Tabak M., Hermens H., Burkow T., Ciobanu I., Berteanu M. Acceptance and usability of an ambulant activity coach for patients with COPD.

[32] Tabak M., Op den Akker H., Hermens H. (2014). Motivational cues as real-time feedback for changing daily activity behavior of patients with COPD. *Patient Education and Counseling*.

